# Variability in the Qualitative and Quantitative Composition of Phenolic Compounds and the *In Vitro* Antioxidant Activity of Sour Cherry (*Prunus cerasus* L.) Leaves

**DOI:** 10.3390/antiox13050553

**Published:** 2024-04-30

**Authors:** Kristina Zymonė, Mindaugas Liaudanskas, Juozas Lanauskas, Miglė Nagelytė, Valdimaras Janulis

**Affiliations:** 1 Institute of Pharmaceutical Technologies, Faculty of Pharmacy, Lithuanian University of Health Sciences, Sukileliu av. 13, LT-50162 Kaunas, Lithuania; 2 Department of Analytical and Toxicological Chemistry, Faculty of Pharmacy, Lithuanian University of Health Sciences, Sukileliu av. 13, LT-50162 Kaunas, Lithuania; 3 Department of Pharmacognosy, Faculty of Pharmacy, Lithuanian University of Health Sciences, Sukileliu av. 13, LT-50162 Kaunas, Lithuania; 4 Institute of Horticulture, Lithuanian Research Centre for Agriculture and Forestry, Kauno Str. 30, LT-54333 Kaunas, Lithuania

**Keywords:** *Prunus cerasus*, phenolic compounds, flavonols, chlorogenic acid, proanthocyanidins, phloridzin, antioxidant activity

## Abstract

Sour cherry (*Prunus cerasus* L.) is a deciduous tree belonging to the *Rosaceae* Juss. family. Cherry leaves are an underutilized source of biologically active compounds. The aim of this study was to determine the composition of the phenolic compounds, as well as the total antioxidant activity, in leaf samples of *P. cerasus* cultivars and to elucidate the cultivars with particular phytochemical compositions. The phytochemical profiles of *P. cerasus* leaves vary significantly in a cultivar-dependent manner. The total content of identified phenolic compounds varied from 8.254 to 16.199 mg/g in the cherry leaves. Chlorogenic acid ranged between 1413.3 µg/g (‘North Star’) and 8028.0 µg/g (‘Note’). The total content of flavonols varied from 4172.5 µg/g (‘Vytenu zvaigzde’) to 9030.7 µg/g (‘Tikhonovskaya’). The total content of identified proanthocyanidins varied from 122.3 µg/g (‘Note’) to 684.8 µg/g (‘Kelleris’). The highest levels of phloridzin (38.1 ± 0.9 µg/g) were found in samples of ‘Molodezhnaya’, while the lowest level of this compound was determined in the leaf samples of ‘Turgenevka’ (6.7 ± 0.2). The strongest antiradical (138.0 ± 4.0 µmol TE/g, *p* < 0.05) and reducing (364.9 ± 10.5 µmol TE/g, *p* < 0.05) activity in vitro was exhibited by the cultivar ‘Vytenu zvaigzde’ cherry leaf sample extracts. ‘Kelleris’, ‘Note’, and ‘Tikhonovskaya’ distinguish themselves with peculiar phytochemical compositions.

## 1. Introduction

Sour cherry (*Prunus cerasus* L.) is a deciduous tree belonging to the *Rosaceae* Juss. family. Sour cherries are native to Europe and Asia and are grown in areas with a temperate climate zone, which are characterized by well-differentiated seasons [1]. The total area covered by sour cherries was 224,425 ha, with the production of fruits amounting to 1,514,664.81 t, in 2021 [2]. These fruits are consumed fresh or processed (juice, preserves, marmalades, jams, drinks, beverages). Besides using them for food, *P. cerasus* fruits are used in traditional medicine [3]. Their positive effect on health (with immunomodulatory [4,5], anti-inflammatory [6,7], antioxidant [8], anticancer [8,9,10,11], antidiabetic [12,13], antimicrobial [14,15,16], gastroprotective [17] activities, skin care, and a protective effect on the skin in terms of UV damage [18]) has also been proven by scientific research.

Biological effects are determined by the phytochemical composition of raw plant materials or the products made from them. The biologically active compounds identified in raw cherry plant materials and the extracts made from them are assigned to various classes. Groups of researchers in different countries have evaluated the phytochemical composition of raw cherry plant materials. Phenolic acids (hydroxybenzoic acids and hydroxycinnamic acids), flavonoids (flavonols, flavonones, flavan-3-ols, anthocyanins), proanthocyanidins, vitamins (vitamin C), minerals, sugars (glucose, fructose, sorbitol, saccharose, galactose, and xylose), organic acids (malic, malonic, oxalic, shikimic, citric, tartaric, quinic, succinic, and fumaric), fatty acids, triterpenic compounds, and amino acids have been found in raw sour cherry plant material [19,20,21,22,23,24,25,26]. Most of these scientific studies have published the results of their research on cherry fruits and their products. On the contrary, the results of phytochemical analyses of the other raw plant materials of sour cherry are fragmented. There is some information on the bioactive compounds in cherry stems [27]; meanwhile, studies comparing the phytochemical composition of sour cherry leaves are lacking. The assessment of the phytochemical composition of raw plant materials is an important stage in the process of searching and screening for potential ingredients for food, cosmetic, or pharmaceutical products. The heterogeneity of plants is common and is used for the selection of garden and medicinal plants, as well as for the evaluation of the quality of raw plant materials. Medicinal plants, fruit trees, and fruit bushes are characterized by interspecific and intraspecific phytochemical diversity. As a result, studies and assessments of their particular phytochemical compositions are very important. Studies of chemical diversity prove the variation in the qualitative and quantitative composition of biologically active compounds between species, varieties, raw plant material, and different plants of the same species. By knowing the phytochemical composition of raw plant material, it is possible to decide about the possibility of not only using the raw material or its extracts, but also of isolating target biologically active compounds containing fractions, which can be selected depending on their biological effect. Phytochemical analysis enables the determination and evaluation of the chemotaxonomic markers of plant species or varieties, which is important for taxonomic classifications—legitimizing, describing and distinguishing species or varieties of plants—and ensuring the quality of healthy, rich in biologically active compounds, and safe food for the consumer.

Cherry leaves are an underutilized source of biologically active compounds. Based on the above-mentioned information about phytochemical compositions and biological effects, it can be concluded that these by-products of sour cherry could be used to create various health-friendly products in the fields of food, cosmetics, and pharmaceuticals.

Cherry leaves could be a sustainable source of food additives, ingredients for functional food, supplements, cosmetic products, and potential pharmaceutical ingredients [28,29]. Natural food additives are intensively searched for as the hazards posed by synthetic additives are identified, some of which are now prohibited to use. Therefore, the interest in natural sources and the development of products with such ingredients is growing. The evaluation of the diverse phytoconstituent compositions of different sour cherry cultivars is a very important step for the evaluation of the quality of *P. cerasus*’s raw plant material. The obtained data can be used in the screening of the most promising cultivars for cultivation and the production or development of products, predicting possible biological effects and possible health-related functions. This information is valuable for producers not only for the standardization of raw plant material, but also for evaluating the quality of the final product and its stability. Despite that, research on the phytochemical composition of sour cherry leaves is still scarce.

The aim of this study was to determine the composition of phenolic compounds and the total antioxidant activity in leaf samples of *P. cerasus* cultivars and to elucidate the cultivars with particular phytochemical compositions. The results of our research provide new, detailed knowledge about the variation of the qualitative and quantitative compositions of phenolic compounds and the antiradical and reductive activity, in vitro, of extracts from sour cherry leaf samples of different cultivars grown in Lithuania, taking into account the influence of the cultivar. The composition of sour cherry leaf samples from most studied cultivars has not been studied at all so far, so the obtained results are important and relevant from analytical, phytochemical, chemotaxonomic, and selection points of view.

## 2. Materials and Methods

### 2.1. Plant Material and Growing Conditions

Sour cherry (*Prunus cerasus* L.) leaves, for a phytochemical analysis, were collected at the Institute of Horticulture, Lithuanian Research Centre for Agriculture and Forestry (55°60′ N, 23°48′ E), in 2020. The leaves were sampled from nursery-grown fruit trees with *P.mahaleb* L. rootstock. Sour cherry cultivars originating from different countries were included in the study: ‘Kelleris’ (Denmark), ‘Lucyna’ (Poland), ‘Pandy’ (Hungary), ‘North Star’ (USA), ‘Vytenu zvaigzde’, ‘Note’ (Lithuania), ‘Molodezhnaya’, ‘Tikhonovskaya’, and ‘Turgenevka’ (Russia). The soil in the fruit tree nursery was Epicalcari–Endohypogleic cambisol, containing 215 mg/kg of P_2_O_5_, 256 mg/kg of K_2_O, and 2.9% of organic matter, pH (1 M KCl)–7.2. The meteorological conditions for fruit tree growth during the April–August period were favorable. According to local iMetos station data, the average temperature was 14.6 °C (https://www.fieldclimate.com/station/0020627F/data; accessed on 21 October 2020), which is 1 °C above the long-term average for the period. April was dry, with only 9.8 mm of rainfall (long-term average 38.4 mm), but this did not adversely affect the fruit trees as the soil retained sufficient moisture reserves from the winter–early spring period. The amount of rainfall in the April–August period was 14% higher than the long-term average. Fruit tree growth was normal.

Leaf samples were taken from the mid-point of lateral shoots on 31 August; raw material was dried at ambient temperature and then stored in a dry, dark place.

### 2.2. Chemicals

All the solvents, reagents, and standards used were of analytical grade. The following substances were used in the study: ethanol 96% (*v*/*v*) (AB “Vilniaus degtinė”, Vilnius, Lithuania), acetic acid (99.8% purity), (“Scharlau”, Sentmenat, Spain), acetonitrile (99.9% purity), chlorogenic acid (95% purity), hyperoside (97% purity), isoquercitrin (90% purity), avicularin (90% purity), rutin (95% purity), procyanidin B1 (90% purity), procyanidin B2 (90% purity), procyanidin C1 (90% purity), phloridzin (98% purity), ABTS 2,2′-Azino-bis(3-ethylbenzothiazoline-6-sulfonic acid) (98% purity), ammonium acetate (98% purity), trolox ((±)-6-Hydroxy-2,5,7,8-tetramethylchromane-2-carboxylic acid) (97% purity), sodium nitrite, sodium molybdate, hydrochloric acid, sodium hydroxide (“Sigma-Aldrich”, Steinheim, Germany), quercitrin (98% purity) (“Extrasynthese”, Lyon, France), reynoutrin (98% purity) (“Cayman Chemical Company”, Ann Arbor, MI, USA), potassium peroxydisuphate (99% purity), neokuproine (98% purity) (“Alfa Aesar Gmbh & Co“, Karlsruhe, Germany), copper (II) chloride dihydrate (99% purity), aluminum (III) chloride, acetic acid, and urotropin (“Carl Roth GmbH + Co. KG”, Karlsruhe, Germany). During the study, we used purified de-ionized water prepared with the “Milli–Q^®^” (“Millipore”, Bedford, MA, USA) water purification system.

### 2.3. Preparation of the Cherry Leaf Sample Extracts

For a quantitative analysis of the phenolic compounds, the cherry leaf samples were crushed to particles that could pass through a 355 μm sieve using an electrical mill “Retsch GM 200” (Retsch GmbH, Haan, Germany) and were stored in tightly closed vessels in a dark and dry place. The ground raw plant materials were weighed using electronic analytical scales “Sartorius CP64–0CE” (“Sartorius AG”, Göttingen, Germany). About a 2 g weight (accurate sample) of raw plant material was placed into a conical flask, into which was poured 15 mL of 40% ethanol. Extraction was performed in a dark place for 72 h. The obtained extract was filtered through a paper filter into a 25 mL volumetric flask. The paper filter was washed 2 times with 5 mL of 40% ethanol. The volume of the obtained extract was adjusted to 25 mL with 40% ethanol. Extracts were filtered through a membrane filter with a pore size of 0.22 μm (Carl Roth GmbH, Karlsruhe, Germany).

### 2.4. Spectrophotometric Techniques

The total content of flavonoids was examined by applying a spectrophotometric method using their reaction with aluminum (III) chloride in an acidic environment. For the preparation of the test sample, 0.1 mL of an ethanolic extract of cherry leaves was used and mixed with 2 mL of 97% (*v*/*v*) ethanol, 0.3 mL of 10% (*v*/*v*) aqueous aluminum chloride solution, 0.1 mL of 30% (*v*/*v*) aqueous acetic acid solution, and 2.1 mL of purified water. Test samples were kept in the dark for 30 min. The samples were then mixed with 0.4 mL of 5% aqueous urotropin solution. For the preparation of the blank sample, 0.1 mL of an ethanolic extract of cherry leaves was used, which was mixed with 2 mL of 97% (*v*/*v*) ethanol, 0.1 mL of 30% (*v*/*v*) aqueous acetic acid, and 2.8 mL of purified water. The absorbances of all the samples were measured with a spectrophotometer at 407 nm. The total content of flavonoids was calculated from the rutin calibration curve and expressed as rutin equivalents in mg/g DW of the leaves.

The total content of hydroxycinnamic acid derivatives was analyzed by applying a spectrophotometric method using their reaction with Arnow’s reagent. The sample was prepared in a 10 mL volumetric flask using 0.1 mL of ethanolic cherry leaf extract diluted with 0.9 mL of 50% (*v*/*v*) ethanol, and the resulting solution was mixed with 2 mL of 8.5% *v*/*v* NaOH, 2 mL 0.5 M HCl, and 2 mL Arnow’s reagent (an aqueous solution of 10% sodium molybdate and 10% sodium nitrite aqueous solution, 1:1). The solution was diluted to the mark with purified water. For the preparation of the blank sample, 0.1 mL of an ethanolic extract of cherry leaves was used, which was mixed in a 10 mL volumetric flask with 0.9 mL of 50% (*v*/*v*) ethanol, 2 mL NaOH, and 2 mL 0.5 M HCl and diluted to the mark with purified water. The absorbances of all the samples were measured with a spectrophotometer at 525 nm. The total content of hydroxycinnamic acid derivatives was calculated from the chlorogenic acid calibration curve and expressed as chlorogenic acid equivalents in mg/g DW of the leaves.

The determination of antioxidant activity was performed using an ABTS·+ radical cation decolorization assay according to the methodology described by Re et al. [30]. A volume of 3 mL of ABTS·+ solution (absorbance 0.8 ± 0.03) was mixed with 3 μL of the ethanol extract of the leaves. A decrease in absorbance was seen at a wavelength of 734 nm after keeping the samples for 60 min in the dark. Antioxidant activity was expressed as Trolox equivalents (TE) in µmol/g DW of the leaves.

The determination of reducing activity was performed using the CUPRAC method described by Apak et al. [31]. A volume of 3 mL of CUPRAC solution was mixed with 5 μL of the ethanol extract of the leaves. A decrease in absorbance was seen at a wavelength of 450 nm after keeping the samples for 30 min in the dark. Antioxidant activity was expressed as Trolox equivalents (TE) in µmol/g DW of the leaves.

### 2.5. Evaluation of Phenolic Compounds in Cherry Leaf Samples Using the HPLC-PDA Technique

The analysis of cherry leaf extracts was performed using the HPLC method. Quantitative analysis was performed using a “Waters 2695 Alliance system” (Waters, Milford, MA, USA) with a photodiode array detector “Waters 2998”. Separation was performed using a YMC-Pack ODS-A (“YMC Europe GmbH”, Germany) column (C18, 250 mm × 4.6 mm, particle size 5 μm) equipped with a YMC-Triart precolumn (C18, 10 mm × 3.0 mm, particle size 5 μm) (“YMC Europe GmbH”, Germany). The mobile phase of the chromatographic method consisted of eluents A (2% acetic acid) and B (acetonitrile). The gradient variation consisted of the following: 0–30 min—3–15% B, 30–45 min—15–25% B, 45–50 min—25–50% B, and 50–55 min—50–95% B. The eluent flow rate was 1.0 mL/min and injection volume 10 μL. The column was temperature-controlled and maintained at 25 °C.

Chromatographic peak identification was carried out according to the analyte and reference compound retention time, as well as by comparing the UV absorption spectra of the reference compounds and analytes obtained with a diode array detector. The contents of phenolic acids were calculated at a wavelength of 320 nm, while the contents of flavonols were calculated at a wavelength of 360 nm. The contents of dihydrochalcones and procyanidins were calculated at a wavelength of 280 nm. The research results were converted to values for absolutely dry plant material.

### 2.6. Statistical Analysis

The content of phenolic compounds is expressed as a mean ± standard error (SE) of three replicates. The statistical data analysis was evaluated by applying an ANOVA with Tukey’s HSD post hoc test. Statistically significant different means were marked with different letters. Differences were considered statistically significant when *p* < 0.05. A principal component analysis was performed. Factors with eigenvalues greater than 1 were considered. A hierarchical cluster analysis was performed using the between-group linkage method with squared Euclidean distances. The data were processed using Microsoft Office Excel for Microsoft 365 MSO (Microsoft, JAV) and IBM SPSS Statistics version 29.0.1.0. software.

## 3. Results and Discussion

### 3.1. Qualitative and Quantitative Composition of the Phenolic Compounds of Sour Cherry (Prunus cerasus L.) Leaves

**Figure 1:**
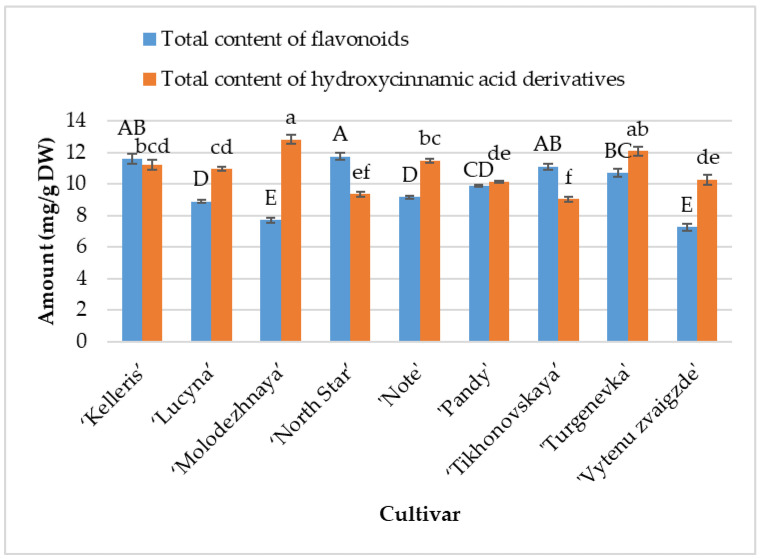
Variation in the total content of flavonoids and total content of hydroxycinnamic acid derivatives (mg/g DW) in sour cherry leaf samples. The averages marked with different letters in the columns show statistically significant differences (at *p* < 0.05).

Spectrophotometric techniques are often applied to determine the quantitative composition of raw plant materials. These methods are used to evaluate the contents of different groups of biologically active compounds. Spectrophotometric methods were applied to determine the variability of the total content of flavonoids in sour cherry leaves, as well as their total content of hydroxycinnamic acid derivatives. The total content of flavonoids varied from 7.27 mg/g to 11.75 mg/g (Figure 1). The highest total contents of flavonoids (*p* < 0.05) were found in the leaf samples of ‘North Star’, ‘Kelleris’, and ‘Tikhonovskaya’ (11.75 ± 0.20 mg/g, 11.61 ± 0.34 mg/g, and 11.12 ± 0.19 mg/g, respectively). The lowest total contents of flavonoids (*p* < 0.05) were determined in the leaf samples of ‘Vytenu zvaigzde’ and ‘Molodezhnaya’ (7.27 ± 0.21 and 7.71 ± 0.18 mg/g, respectively). Similar total contents of flavonoids were found in the methanolic extracts of *P. persica* (L.) Batsch leaves (649.25–1011.26 mg/100 g dw) [32] and the methanolic extracts of *P. scoparia* (Spach) C.K. Schneid. leaves (8.34 to 11.32 mg/g dw) [33].

The total content of hydroxycinnamic acid derivatives ranged between 9.03 mg/g and 12.82 mg/g (Figure 1). The highest total contents of hydroxycinnamic acid derivatives (*p* < 0.05) were determined in the leaf samples of ‘Molodezhnaya’ and ‘Turgenevka’ (12.82 ± 0.30 mg/g and 12.10 ± 0.28 mg/g, respectively). The lowest total contents of hydroxycinnamic acid derivatives (*p* < 0.05) were found in the leaf samples of ‘Tikhonovskaya’ and ‘North Star’ (9.03 ± 0.16 mg/g, and 9.35 ± 0.16 mg/g, respectively).

Phenolic acid (chlorogenic acid), flavonols (rutin, hyperoside, isoquercitrin, reynoutrin, avicularin, quercitrin), oligomeric flavan-3-ols (procyanidin B1, procyanidin B2, procyanidin C1), and dihydrochalcone phloridzin were identified in cherry leaf samples when applying the HPLC-PDA method. These findings are in agreement with the results published by other scientists. Other scientific groups have identified biologically active compounds belonging to the same groups. According to Nowak et al., epigallocatechin, quercetin glucoside, and kaempferol glucoside can be identified in *P. cerasus* extracts [23]. Chrzanowski et al. stated that sour cherry leaves accumulate phenolic acids, including chlorogenic acid [25]. Similar groups of biologically active compounds have been identified in *P. cerasus* fruit extracts [11].

The total content of identified phenolic compounds varied from 8.254 to 16.199 mg/g in cherry leaves. The total content of phenolic compounds in the water extracts of cherry leaf samples was evaluated by Nowak et al. and reached 3.17 mg GAE/g [23]. Wojdylo et al. studied the phytochemical composition of sour cherry extracts (the extrahent was water/methanol/ascorbic acid/37% hydrochloric acid 6.8:3:0.1:0.1) and determined that there were 1584.8–2955.6 mg/100 g dw of phenolic compounds in sour cherry leaves [24]. It is worth noting that the raw plant material of cherry leaves is richer in phenolic compounds than the fruits of sour cherry. Wojdylo et al. found lower contents of phenolic compounds in cherry fruit (665.8–871.7 mg/100 g dw) [24]. Even lower contents of total phenolic compounds (96.56–312.0 mg/100 g FW) were quantified in fresh fruit samples by other groups of scientists [20,34]. Brozdowski et al. analyzed the phenolic profile of extracts of *P. serotine* Ehrh. leaf samples [35]. This scientific group found that the total contents of analyzed phenolic compounds in their methanolic and water extracts were 36,512 ± 3596 mg/kg dw and 28,318 ± 2806 mg/kg dw.

The coefficients of variation of the total content of identified phenolic compounds varied from 30.4 to 117.6%. The high values determined for this coefficient show the important impact of cultivars on the phytochemical composition of cherry leaves. This is confirmed by other published research results which identify this factor as one of the most important factors determining the composition of raw plant materials, along with factors such as maturity, agronomic factors, and climatic conditions [20].

Chlorogenic acid is a common component in the phytochemical profiles of raw plant materials. This compound not only has research-based beneficial effects on health (antibacterial [36], antiviral [37], antitumor [38], anti-inflammatory [39] activities, regulation of glucose [40] and lipid [41] metabolism, protection of the nervous system [42], liver [43], and kidney [44], and a positive effect for those suffering from cardiovascular diseases [45]), but it can be successfully applied in the food industry (including as food additives (emulsifiers, colorants, preservatives), in food storage, food composition modification, food packaging materials, functional food materials, and prebiotics) [46]. Moreover, this hydroxycinnamic acid is a strong antioxidant with anti-aging and photoprotecting effects and can be used in the development of cosmetic products [47].

Chlorogenic acid was the predominant compound in the complex of identified phenolics in all leaf samples except the samples of ‘North Star’ and ‘Tikhonovskaya’. The determined content of chlorogenic acid was 15.00–55.10% of the total content of identified phenolic compounds. Caffeoylquinic acids were quantified as the major constituents by other authors [24,26]. These biologically active compounds were identified as the predominant components in the complex of phenolic acids in the leaf samples of other *Prunus* species [35,48,49]. The chlorogenic acid in cherry leaf samples ranged between 1413.3 µg/g (in leaf samples of ‘North Star’) and 8028.0 µg/g (in leaf samples of ‘Note’) (Table 1). The determined content of chlorogenic acid corresponds to previously published results (up to 3.792 ± 0.040 mg/g of chlorogenic acid in cherry leaves) [25,26]. Our previously studied apple leaf samples showed a lower amount of chlorogenic acid (0.48–1.38 mg/g) than these cherry leaf samples [50].

Furthermore, flavonols are promising compounds with wide-ranging application possibilities in the fields of textiles (as agents for chemical processing, functionalization, dyeing, and the production of fibers), cosmetics (due to their photoprotecting, anti-aging, hyperpigmentation protecting, and collagenase-, elastase-, and tyrosinase-inhibiting activity) and the food industry (as preservatives, shelf-life enhancers, and ingredients for food supplements and functional food) [51]. The total content of flavonols varied from 4172.5 µg/g (‘Vytenu zvaigzde’) to 9030.7 µg/g (‘Tikhonovskaya’). A similar content of flavonoids in cherry leaf samples has been found in previous studies (292.4–1551.6 mg/100 DW) [24]. Quercitrin was the main compound in the complex of identified flavonols in the leaves of all cultivars except the leaf samples of ‘Kelleris’ and ‘Molodezhnaya’. Moreover, this flavonol was the predominant phytoconstituent in the leaf samples of ‘North Star’ and ‘Tikhonovskaya’. The determined content of quercitrin was 13.99–59.52% of the total content of identified phenolic compounds. The quercitrin in cherry leaf samples ranged between 1933.9 µg/g (in leaf samples of ‘Kelleris’) and 5610.0 µg/g (in leaf samples of ‘North Star’) (Table 2). The leaves of ‘Molodezhnaya’ were characterized by the highest levels of avicularin (2194.0 ± 50.7 µg/g). Avicularin was the main flavonoid in the leaf samples of black cherries [35]. In the leaves of ‘Kelleris’, the main component of the flavonol complex was isoquercitrin (2635.4 ± 76.1 µg/g) (Table 2). The highest content of hyperoside (1815.5 ± 52.4 µg/g) was also determined in these samples (Table 2).

Rutin and reynoutrin made up 0.75 and 1.50% of all identified phenolics, respectively, and could be described as minor constituents. Contrary to our results, the glucosides and rutinosides quercetin, kaempferol, and isorhamnetin were the predominant compounds in the complex of flavonols in cherry fruit samples [24,52]. In addition to this, rutin was one of the main components in leaf samples of *P. domestica* L. [49]. The obtained research results were compared with the data on the analysis of the phenolic compounds in our previously studied apple leaf samples. Quercitrin also prevailed in apple leaf samples, while a higher quercitrin content (7.77–13.36 mg/g) was found than that in cherry leaf samples. Likewise, higher amounts of rutin (0.33–0.75 mg/g) and hyperoside (4.59–8.95 mg/g) were found in the apple leaf samples than in the cherry leaf samples of the cultivars we studied [50].

The results of published studies show the potential of proanthocyanidins to be applied to achieve hypoglycemic [53], hypolipidemic [54], cardioprotective [55], neuroprotective [56], immunomodulatory [57], and intestinal-flora-regulating [58] effects. In addition to their beneficial health effects, proanthocyanidins are widely used in the food industry (as natural dyes and preservatives). These compounds are promising ingredients for cosmeceutical products, as several studies have revealed their whitening, anti-aging, anti-sun, and moisturizing activity [59].

Proantocyanidins made up 3.07% of all identified phenolic compounds when evaluating each compound separately in the profiles of the cherry leaves. The total content of identified proanthocyanidins varied from 122.3 µg/g (‘Note’) to 684.8 µg/g (‘Kelleris’). Wojdyło et al. determined a higher content of flavanols (905.1–1106.2 mg/100 g dw) in cherry leaf samples [24].

The greatest amounts of procyanidin B2 were determined in samples of ‘Vytenu zvaigzde’ (138.4 ± 4.0 µg/g), while the highest levels of procyanidin B1 and procyanidin C1 were found in leaves of ‘Kelleris’ (370.4 ± 10.7 and 209.0 ± 6.0, respectively) (Table 1).

Dihydrochalcone phloridzin was also a minor component in the profile of cherry leaves. Besides its health benefits (anti-cancer, anti-obesity, anti-diabetic, antioxidant, anti-ageing, antimicrobial, and melanogenic activity [60,61]), dihydrochalcone phloridzin can be used as a natural substitute for tartrazine [62]. Moreover, the results of different studies indicate the potential application of phloretin (aglycone of phloridzin) in the cosmetic industry due to its established antioxidant, antiaging, and depigmenting effects [63]. The highest levels of phloridzin (38.1 ± 0.9 µg/g) were found in samples of ‘Molodezhnaya’ (Table 1). Significantly higher levels of phloridzin (106–114 mg/g) were found in apple leaf samples than in the cherry leaf samples we studied [50].

**Figure 2:**
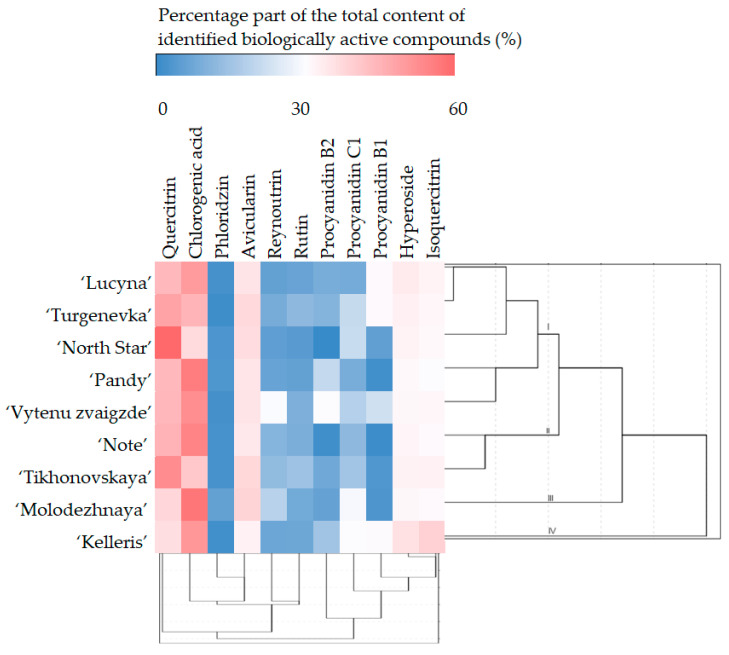
Dendrogram of the hierarchical cluster analysis of sour cherry leaf samples according to their phytochemical composition and a heatmap of the percentage compositions of their identified phenolic compounds.

A hierarchical cluster analysis was performed on the cherry leaf samples, using the amounts of the identified biologically active compounds as clustering variables. The investigated samples of cherry leaves were grouped into four significant clusters (Figure 2).

The first cluster grouped the leaf samples of ‘Lucyna’, ‘Turgenevka’, ‘North Star’, ‘Pandy’, and ‘Vytenu zvaigzde’. In the leaf samples of these cultivars, the content of their identified biologically active compounds was close to the mean values. The leaf samples of ‘Note’ and ‘Tikhonovskaya’ formed the second cluster. The samples forming this cluster were characterized by high contents of quercitrin, avicularin, reynoutrin, and rutin and low contents of procyanidin B1 and procyanidin B2. The leaves of ‘Molodezhnaya’ and ‘Kelleris’ formed the third and fourth clusters, respectively. Leaves of the ‘Molodezhnaya’ cultivar differed from the others in having high contents of chlorogenic acid, avicularin, reynoutrin, phloridzin, and procyanidin C1 and low contents of quercitrin, hyperozide, isoquercitrin, procyanidin B1, and procyanidin B2, while the leaves of ‘Kelleris’ were characterized by high contents of chlorogenic acid, hyperozide, isoquercitrin, procyanidin B1, procyanidin B2, and procyanidin C1 as well as low contents of quercitrin and avicularin.

**Figure 3:**
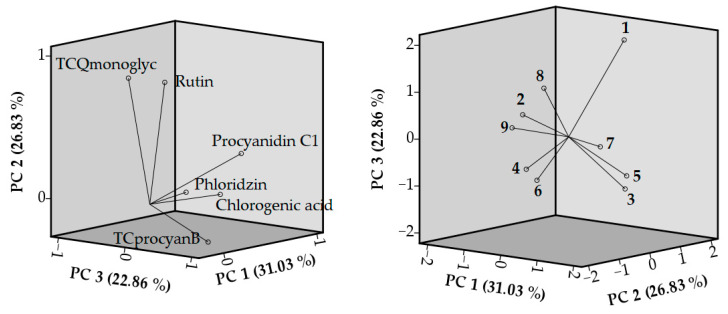
PCA loading plots and score plots of different cherry leaf samples. PC—principal component; TCQmonoglyc—the total content of quercetin monoglycosides; TCprocyanB—the total content of identified B type proanthocyanidines. 1—‘Kelleris’; 2—‘Lucyna’; 3—‘Molodezhnaya’; 4—‘North Star’; 5—‘Note’; 6—‘Pandy’; 7—‘Tikhonovskaya’; 8—‘Turgenevka’; 9—‘Vytenu zvaigzde’.

A principal component analysis (PCA) was applied to detect the similarities and differences between the analyzed cherry leaf samples according to their total content of quercetin monoglycosides (TCQmonoglyc), their rutin content, their total identified B type proanthocyanidines (TCprocyanB) content, their procyanidin C1 content, their phloridzin content, and their chlorogenic acid content. The PCA results are shown in Figure 3. Three principal components explaining 80.72% of the total data variance in the data sets of the cherry leaves were used for the in-depth analysis. Principal component 1 (PC1) described 31.32% of the total variance of the data and correlated with positive loadings of chlorogenic acid (0.827), phloridzin (0.781), and procyanidin C1 (0.710). Principal component 2 (PC2) described 26.83% of the total variance and was characterized by positive loadings of TCQmonoglyc (0.897) and rutin (0.825). Principal component 3 (PC3) accounted for 22.86% of the total variance of the data and had a very strong positive correlation with TCprocyanB (0.951).

PC1 differentiates the leaf samples containing the highest levels of chlorogenic acid, phloridzin, and procyanidin C1. Leaf samples of ‘Molodezhnaya’ were distanced from all the others and were grouped on the positive side of PC1. These leaf samples contained the highest contents of phloridzin. Their contents of chlorogenic acid and procyanidin C1 were higher than the average contents of these biologically active compounds in the cherry leaf samples. On the other hand, a negative correlation to PC2 and PC3 was determined due to the low content of rutin and low total contents of identified quercetin monoglycosides and B type proanthocyanidins. Leaf samples of ‘Note’ and ‘Kelleris’ were distinguished by their high contents of chlorogenic acid, procyanidin C1, rutin, and total content of identified monoglycosides of quercetin, leading to their position on the positive side of PC 1 and PC2. Meanwhile, leaf samples of ‘Kelleris’ were distanced from the others on the positive side of PC3. These leaf samples contained the highest levels of the total content of identified B type proanthocyanidins. PC2 marks out the leaf samples from ‘Tikhonovskaya’. We determined the highest levels of rutin and total contents of quercetin monoglycosides in these leaf samples. The leaf samples of other cultivars were located near the zero point or on the negative sides of the used principal components. The phytochemical compositions of these leaf samples were similar, and the contents of their compounds were close to or lower than the mean values.

The results of the phytochemical analysis revealed the important impact of cultivars. This is confirmed by the data published by other researchers. The differences in the phytochemical compositions obtained by different scientific groups can also be explained by the influence of other factors. Based on the results presented by other research groups, it can be concluded that the phytochemical composition depends also on vegetation stages, climate, edaphic factors, and cultivation methods [64,65,66,67]. Furthermore, storage conditions and processing methods affect the quality not only of the raw plant material but also of the product made from it [68,69,70].

### 3.2. Determination of In Vitro Antioxidant Activity of Cherry Leaf Extracts

After determining the variation in the qualitative and quantitative composition of the phenolic compounds of cherry leaf samples of different varieties, it is important to study and evaluate the antioxidant activity of cherry leaf sample extracts in vitro. The obtained research results could be potentially valuable for using the plant raw material of cherry leaves for the development of products rich in natural antioxidants, their standardization and quality control, and for predicting the antioxidant activity of cherry leaves in vivo.

Plant extracts are multicomponent matrices whose antioxidant activity occurs through several different reaction mechanisms [71,72]. For this reason, it is recommended that at least two methodologies with different mechanisms be applied to studies of plant extracts [73]. The ABTS radical–cation binding methodology was applied to evaluate the antiradical activity of sample extracts of cherry leaves in vitro, and their reducing activity in vitro was determined by applying the CUPRAC methodology.

**Figure 4:**
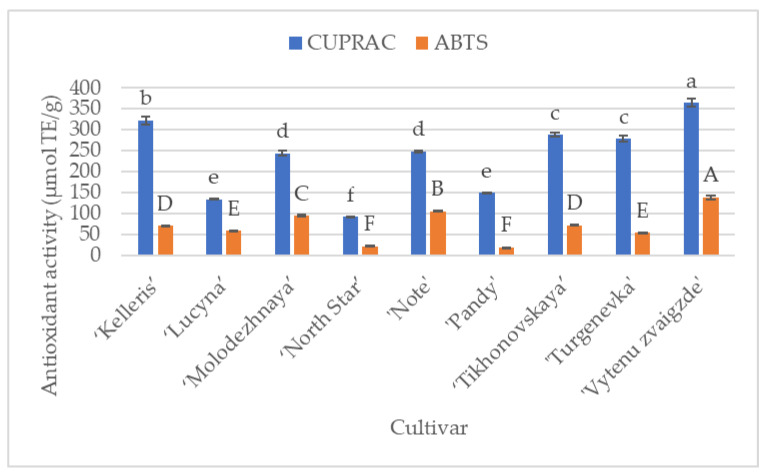
Antioxidant activity (µmol TE/g) of sour cherry leaf samples. Averages marked with different letters in their columns show statistically significant differences (at *p* < 0.05).

The results of the cherry leaf samples’ antioxidant activity study revealed that, statistically significantly, the strongest antiradical (138.0 ± 4.0 µmol Trolox equivalents (TE)/g, *p* < 0.05) and reducing (364.9 ± 10.5 µmol TE/g, *p* < 0.05) activity in vitro were exhibited by the cherry leaf sample extracts of the cultivar ‘Vytenu zvaigzde’. The weakest antiradical activity was determined after examining the extracts of ‘North Star’ and ‘Pandey’ cherry leaf samples (21.62 ± 0.4 µmol TE/g and 18.87 ± 0.1 µmol TE/g, respectively). Extracts from cherry leaf samples of the ‘Lucyna’ and ‘Pandey’ cultivars showed the weakest reducing activity (133.40 ± 1.5 µmol TE/g and 149.04 ± 0.9 µmol TE/g, respectively) (Figure 4).

Sokół-Łętowska et al. measured the antioxidant activity of fresh cherry fruits using DPPH and FRAP methods. The determined antioxidant activity was 510.6–984.8 µmol TE/100 g FW and 1111.1–3065.8 µmol TE/100 g FW, respectively [20]. Comparing the results of our research with the data from other *in vitro* antioxidant activity studies of leaf extracts of the Rosaceae family that applied the ABTS method shows that the *in vitro* antiradical activity of cherry leaf sample extracts is weaker than that of apple (280.23 µmol TE/g) and rowan (262.5–467.3 µmol/g) leaf sample extracts [50,74].

The coefficients of variation were calculated to assess the amplitude of the variation of the antiradical and reducing activity of cherry leaf extracts. A significant variation in the antioxidant activity of the tested extracts in vitro was shown. The coefficient of variation of their reducing activity reached 39%, and that of their antiradical activity reached 55.1%. Such results could have been influenced by the cultivar factor, which is indicated by our work, as well as other authors’ previous studies, on the in vitro antioxidant activity of the leaves and fruits of plants of the Rosaceae family [50,75].

## 4. Conclusions

Sour cherry leaves can be regarded as a promising source of phenolic compounds. The phytochemical profiles of *P. cerasus* leaves vary significantly in a cultivar-dependent manner. Their phenolic fingerprint profiles can be used as markers for assessing the identity and quality of their raw materials and products. After the evaluation of the qualitative and quantitative compositions of the phenolic compounds in the samples of sour cherry leaves of different varieties, the identified analytical markers (chlorogenic acid and quercitrin) are promising for the evaluation of the composition of cherry leaves and the functional food, food supplements, and other preparations made from them, to provide the consumer with high-quality, biologically known products for the composition of active compounds. ‘Kelleris’, ‘Note’, and ‘Tikhonovskaya’ distinguish themselves with particular phytochemical compositions and could be target genotypes for in-depth studies on phytochemical compositions, fractionating target compounds, or assessing the possibilities of their application in innovative food, feeds, and cosmetics production. During the conducted research, the sour cherry cultivars whose leaf samples accumulate the highest amounts of phenolic compounds—natural antioxidants—and whose extracts exhibit the strongest antiradical and reducing activity in vitro were identified. The sour cherry leaves of these cultivars are low-cost, accessible, and potentially valuable for further in vitro and in vivo studies, predicting the in vivo antioxidant effects of sample cherry leaf extracts, the isolation of individual phenolic compounds (especially chlorogenic acid and quercitrin), various dietary supplements or functional foods enriched with cherry leaves, or the development and production of their extracts.

## Figures and Tables

**Table 1:** Contents of chlorogenic acid, phloridzin, and procyanidins (µg/g DW) in sour cherry leaf samples.

Cultivar	Compound				
	Chlorogenic Acid	Phloridzin	Procyanidin B2	Procyanidin C1	Procyanidin B1
‘Kelleris’	5809.6 ± 167.7c ^1^	13.4 ± 0.4de	105.4 ± 3.0c	209.0 ± 6.0a	370.4 ± 10.7a
‘Lucyna’	4427.8 ± 51.1d	12.8 ± 0.1e	50.2 ± 0.6d	47.7 ± 0.6e	333.2 ± 3.8b
‘Molodezhnaya’	6751.4 ± 155.9b	38.1 ± 0.9a	38.4 ± 0.9e	179.0 ± 4.1b	19.1 ± 0.4e
‘North Star’	1413.3 ± 24.5g	15.5 ± 0.3c	2.6 ± 0.0g	102.2 ± 1.8c	27.0 ± 0.5e
‘Note’	8028.0 ± 92.7a	20.6 ± 0.2b	12.9 ± 0.1f	99.9 ± 1.2c	9.5 ± 0.1e
‘Pandy’	6130.7 ± 35.4c	20.1 ± 0.1b	123.4 ± 0.7b	54.2 ± 0.3e	11.4 ± 0.1e
‘Tikhonovskaya’	2702.3 ± 46.8f	15.1 ± 0.3cd	47.1 ± 0.8de	93.2 ± 1.6c	21.9 ± 0.4e
‘Turgenevka’	2825.1 ± 65.2f	6.7 ± 0.2g	50.9 ± 1.2d	99.3 ± 2.3c	282.6 ± 6.5c
‘Vytenu zvaigzde’	3763.1 ± 108.6e	8.6 ± 0.2f	138.4 ± 4.0a	77.1 ± 2.2d	94.4 ± 2.7d

^1^ Averages marked with different letters in their columns show statistically significant differences (at *p* < 0.05).

**Table 2:** Contents of flavonols (µg/g DW) in sour cherry leaf samples.

Cultivar	Compound					
	Rutin	Reynoutrin	Quercitrin	Isoquercitrin	Avicularin	Hyperoside
‘Kelleris’	52.8 ± 1.5cd ^1^	51.3 ± 1.5d	1933.9 ± 55.8e	2635.4 ± 76.1a	829.0 ± 23.9f	1815.5 ± 52.4a
‘Lucyna’	36.8 ± 0.4e	33.1 ± 0.4ef	3135.2 ± 36.2c	567.3 ± 6.6c	1268.0 ± 14.6e	945.8 ± 10.9b
‘Molodezhnaya’	51.4 ± 1.2d	117.1 ± 2.7a	2084.1 ± 48.1e	341.7 ± 7.9ef	2194.0 ± 50.7a	438.5 ± 10.1ef
‘North Star’	21.6 ± 0.4f	26.5 ± 0.5f	5610.0 ± 97.2a	306.8 ± 5.3ef	1396.6 ± 24.2de	502.8 ± 8.7e
‘Note’	77.4 ± 0.9b	89.2 ± 1.0b	5048.3 ± 58.3b	477.2 ± 5.5cd	1611.5 ± 18.6c	724.2 ± 8.4c
‘Pandy’	35.6 ± 0.2e	40.1 ± 0.2e	3319.1 ± 19.2c	243.1 ± 1.4f	1312.1 ± 7.6e	402.7 ± 2.3f
‘Tikhonovskaya’	89.2 ± 1.5a	78.4 ± 1.4c	5501.3 ± 95.3a	701.0 ± 12.1b	1965.2 ± 34.0b	695.6 ± 12.0cd
‘Turgenevka’	57.6 ± 1.3c	41.0 ± 0.9e	3466.7 ± 80.1c	388.9 ± 9.0de	1473.5 ± 34.0cd	605.4 ± 14.0d
‘Vytenu zvaigzde’	40.3 ± 1.2e	123.8 ± 3.6a	2416.3 ± 69.8d	347.4 ± 10.0def	944.8 ± 27.3f	299.8 ± 8.7g

^1^ Averages marked with different letters in their columns show statistically significant differences (at *p* < 0.05).

## Data Availability

Data contained within the article.

## References

[1] Serradilla M.J., Hernández A., López-Corrales M., Ruiz-Moyano S., de Guía Córdoba M., Martín A. (2015). Composition of the Cherry (*Prunus avium* L. and *Prunus cerasus* L.; Rosaceae). Nutritional Composition of Fruit Cultivars.

[2] FAOSTAT. https://www.fao.org/faostat/en/#data/QCL.

[3] Ahmad I., Shamsi S., Zaman R. (2017). A Review on Sour Cherry (*Prunus cerasus*): A High Value Unani Medicinal Fruit. Int. J. Green Pharm..

[4] Abid S., Khajuria A., Parvaiz Q., Sidiq T., Bhatia A., Singh S., Ahmad S., Randhawa M.K., Satti N.K., Dutt P. (2012). Immunomodulatory Studies of a Bioactive Fraction from the Fruit of *Prunus cerasus* in BALB/c Mice. Int. Immunopharmacol..

[5] Ali S.A., Ahmad R., Ahmad N., Makhdoomi M., Parvaiz Q. (2019). Augmentation of Immunocytes Functions by *Prunus cerasus* Fruit and Its Biotherapeutic Potential in Mice Model. Biomed. Pharmacol. J..

[6] Biro A., Markovich A., Homoki J.R., Szollosi E., Hegedus C., Tarapcsák S., Lukács J., Stündl L., Remenyik J. (2019). Anthocyanin-Rich Sour Cherry Extract Attenuates the Lipopolysaccharide-Induced Endothelial Inflammatory Response. Molecules.

[7] Šarić A., Sobočanec S., Balog T., Kušić B., Šverko V., Dragović-Uzelac V., Levaj B., Čosić Z., Šafranko Ž.M., Marotti T. (2009). Improved Antioxidant and Anti-Inflammatory Potential in Mice Consuming Sour Cherry Juice (*Prunus cerasus* Cv. Maraska). Plant Foods Hum. Nutr..

[8] Nikolaou E.N., Karvela E.D., Papadopoulou A., Karathanos V.T. (2023). The Effect of Enrichment with Sour-Cherry Extracts on Gluten-Free Snacks Developed by Novel 3D Technologies. Antioxidants.

[9] Khoo G.M., Clausen M.R., Pedersen B.H., Larsen E. (2012). Bioactivity of Sour Cherry Cultivars Grown in Denmark. Phytother. Res..

[10] Maragheh A.D., Tabrizi M.H., Karimi E., Seyedi S.M.R., Khatamian N. (2019). Producing the Sour Cherry Pit Oil Nanoemulsion and Evaluation of Its Anti-Cancer Effects on Both Breast Cancer Murine Model and MCF-7 Cell Line. J. Microencapsul..

[11] Sheikh A.A., Wani Z.A., Shah A.M., Hassan Q.P., Mondhe D.M., Verma M.K. (2022). Chemopreventive Effects of *Prunus cerasus* L. against Human Cancer Cells & Ascites Mice Models and Its Phytochemical Investigation by LC-Q-TOF-MS/MS. Phytomedicine Plus.

[12] Saleh F.A., El-Darra N., Raafat K. (2017). Hypoglycemic Effects of *Prunus cerasus* L. Pulp and Seed Extracts on Alloxan-Induced Diabetic Mice with Histopathological Evaluation. Biomed. Pharmacother..

[13] Xiao G., Xiao X. (2019). Antidiabetic Effect of Hydro-Methanol Extract of *Prunus cerasus* L. Fruits and Identification of Its Bioactive Compounds. Trop. J. Pharm. Res..

[14] Ben Lagha A., LeBel G., Grenier D. (2020). Tart Cherry (*Prunus cerasus* L.) Fractions Inhibit Biofilm Formation and Adherence Properties of Oral Pathogens and Enhance Oral Epithelial Barrier Function. Phytother. Res..

[15] Berroukche A., Benreguieg M., Terras M., Fares S., Dellaoui H., Lansari W., Zerarki I., Tahir A., Dehkal B. (2018). Antibacterial Effects of Prunus cerasus and Chamaemelum Nobile against Drug Resistant Strains Induced Urinary Disorders. East Afr. Sch. J. Med. Sci..

[16] Krisch J., Galgoczy L., Papp T., Vágvölgyi C., Galgóczy L. (2009). Antimicrobial and Antioxidant Potential of Waste Products Remaining after Juice Pressing. Ann. Fac. Eng. Hunedoara-J. Eng..

[17] Raafat K., El-Darra N., Saleh F.A. (2020). Gastroprotective and Anti-Inflammatory Effects of *Prunus cerasus* Phytochemicals and Their Possible Mechanisms of Action. J. Tradit. Complement. Med..

[18] Bak I., Czompa A., Csepanyi E., Juhasz B., Kalantari H., Najm K., Aghel N., Varga B., Haines D.D., Tosaki A. (2011). Evaluation of Systemic and Dermal Toxicity and Dermal Photoprotection by Sour Cherry Kernels. Phytother. Res..

[19] Yılmaz F.M., Görgüç A., Karaaslan M., Vardin H., Ersus Bilek S., Uygun Ö., Bircan C. (2019). Sour Cherry By-Products: Compositions, Functional Properties and Recovery Potentials—A Review. Crit. Rev. Food Sci. Nutr..

[20] Sokół-Łętowska A., Kucharska A.Z., Hodun G., Gołba M. (2020). Chemical Composition of 21 Cultivars of Sour Cherry (*Prunus cerasus*) Fruit Cultivated in Poland. Molecules.

[21] Stryjecka M., Michalak M., Cymerman J., Kiełtyka-Dadasiewicz A. (2022). Comparative Assessment of Phytochemical Compounds and Antioxidant Properties of Kernel Oil from Eight Sour Cherry (*Prunus cerasus* L.) Cultivars. Molecules.

[22] Chatzimitakos T., Athanasiadis V., Kalompatsios D., Kotsou K., Mantiniotou M., Bozinou E., Lalas S.I. (2024). Sustainable Valorization of Sour Cherry (*Prunus cerasus*) By-Products: Extraction of Antioxidant Compounds. Sustainability.

[23] Nowak A., Czyzowska A., Efenberger M., Krala L. (2016). Polyphenolic Extracts of Cherry (*Prunus cerasus* L.) and Blackcurrant (*Ribes nigrum* L.) Leaves as Natural Preservatives in Meat Products. Food Microbiol..

[24] Wojdyło A., Nowicka P., Turkiewicz I.P., Tkacz K. (2021). Profiling of Polyphenols by LC-QTOF/ESI-MS, Characteristics of Nutritional Compounds and In Vitro Effect on Pancreatic Lipase, α-Glucosidase, α-Amylase, Cholinesterase and Cyclooxygenase Activities of Sweet (*Prunus avium*) and Sour (*P. cerasus*) Cherries Leaves and Fruits. Ind. Crops Prod..

[25] Chrzanowski G., Sempruch C., Sprawka I. (2007). Investigation of Phenolic Acids in Leaves of Black Currant (*Ribes nigrum* L.) and Sour Cherry (*Prunus cerasus* L.) Molecular Diagnostics of Pathogens Transmitted by Ixodes Ricinus Ticks View Project Mealybugs-Orchid Interactions View Project. https://www.researchgate.net/publication/262696254.

[26] Oszmiański J., Wojdyło A. (2014). Influence of Cherry Leaf-Spot on Changes in the Content of Phenolic Compounds in Sour Cherry (*Prunus cerasus* L.) Leaves. Physiol. Mol. Plant Pathol..

[27] Švarc-Gaji J., Cerdà V., Clavijo S., Suárez R., Maškovi P., Cvetanovi A., Delerue-Matos C., Carvalho A.P., Novakov V. (2017). Bioactive Compounds of Sweet and Sour Cherry Stems Obtained by Subcritical Water Extraction. J. Chem. Technol. Biotechnol..

[28] Efenberger-Szmechtyk M., Nowak A., Czyzowska A. (2021). Plant Extracts Rich in Polyphenols: Antibacterial Agents and Natural Preservatives for Meat and Meat Products. Crit. Rev. Food Sci. Nutr..

[29] Mutha R.E., Tatiya A.U., Surana S.J. (2021). Flavonoids as Natural Phenolic Compounds and Their Role in Therapeutics: An Overview. Futur. J. Pharm. Sci..

[30] Re R., Pellegrini N., Proteggente A., Pannala A., Yang M., Rice-Evans C. (1999). Antioxidant Activity Applying an Improved ABTS Radical Cation Decolorization Assay. Free Radic. Biol. Med..

[31] Apak R., Güçlü K., Demirata B., Özyürek M., Çelik S.E., Bektaşoğlu B., Berker K.I., Özyurt D. (2007). Comparative Evaluation of Various Total Antioxidant Capacity Assays Applied to Phenolic Compounds with the CUPRAC Assay. Molecules.

[32] Maatallah S., Dabbou S., Castagna A., Guizani M., Hajlaoui H., Ranieri A.M., Flamini G. (2020). Prunus Persica By-Products: A Source of Minerals, Phenols and Volatile Compounds. Sci. Hortic..

[33] Taati S., Pilehvar B., Mirazadi Z. (2022). Essential Oil, Phenol and Flavonoid Contents in Leaves and Fruits of Prunus Scoparia (Spach) C.K. Schneid. Populations. J. Med. Plants By-Prod..

[34] Levaj B., Dragović-Uzelac V., Delonga K., Kovačević Ganić K., Banović M., Bursać Kovačević D. (2010). Polyphenols and Volatiles in Fruits of Two Sour Cherry Cultivars, Some Berry Fruits and Their Jams. Food Technol. Biotechnol..

[35] Brozdowski J., Waliszewska B., Gacnik S., Hudina M., Veberic R., Mikulic-Petkovsek M. (2021). Phenolic Composition of Leaf and Flower Extracts of Black Cherry (*Prunus serotina* Ehrh.). Ann. Sci..

[36] Lou Z., Wang H., Zhu S., Ma C., Wang Z. (2011). Antibacterial Activity and Mechanism of Action of Chlorogenic Acid. J. Food Sci..

[37] Wang G.F., Shi L.P., Ren Y.D., Liu Q.F., Liu H.F., Zhang R.J., Li Z., Zhu F.H., He P.L., Tang W. (2009). Anti-Hepatitis B Virus Activity of Chlorogenic Acid, Quinic Acid and Caffeic Acid In Vivo and In Vitro. Antivir. Res..

[38] Zeng A., Liang X., Zhu S., Liu C., Wang S., Zhang Q., Zhao J., Song L. (2021). Chlorogenic Acid Induces Apoptosis, Inhibits Metastasis and Improves Antitumor Immunity in Breast Cancer via the NF-ΚB Signaling Pathway. Oncol. Rep..

[39] David dos Santos M., Camila Almeida M., Peporine Lopes N., Emília Petto de Souza G. (2006). Evaluation of the Anti-Inflammatory, Analgesic and Antipyretic Activities of the Natural Polyphenol Chlorogenic Acid. Biol. Pharm. Bull..

[40] Bassoli B.K., Cassolla P., Borba-Murad G.R., Constantin J., Salgueiro-Pagadigorria C.L., Bazotte R.B., Da Silva R.S.D.S.F., De Souza H.M. (2008). Chlorogenic Acid Reduces the Plasma Glucose Peak in the Oral Glucose Tolerance Test: Effects on Hepatic Glucose Release and Glycaemia. Cell Biochem. Funct..

[41] Karthikesan K., Pari L., Menon V.P. (2010). Antihyperlipidemic Effect of Chlorogenic Acid and Tetrahydrocurcumin in Rats Subjected to Diabetogenic Agents. Chem. Biol. Interact..

[42] Nabavi S.F., Tejada S., Setzer W.N., Gortzi O., Sureda A., Braidy N., Daglia M., Manayi A., Nabavi S.M. (2016). Chlorogenic Acid and Mental Diseases: From Chemistry to Medicine. Curr. Neuropharmacol..

[43] Shi H., Shi A., Dong L., Lu X., Wang Y., Zhao J., Dai F., Guo X. (2016). Chlorogenic Acid Protects against Liver Fibrosis In Vivo and in Vitro through Inhibition of Oxidative Stress. Clin. Nutr..

[44] Domitrović R., Cvijanović O., Šušnić V., Katalinić N. (2014). Renoprotective Mechanisms of Chlorogenic Acid in Cisplatin-Induced Kidney Injury. Toxicology.

[45] Bhandarkar N.S., Brown L., Panchal S.K. (2019). Chlorogenic Acid Attenuates High-Carbohydrate, High-Fat Diet–Induced Cardiovascular, Liver, and Metabolic Changes in Rats. Nutr. Res..

[46] Wang L., Pan X., Jiang L., Chu Y., Gao S., Jiang X., Zhang Y., Chen Y., Luo S., Peng C. (2022). The Biological Activity Mechanism of Chlorogenic Acid and Its Applications in Food Industry: A Review. Front. Nutr..

[47] Rodrigues R., Oliveira M.B.P.P., Alves R.C. (2023). Chlorogenic Acids and Caffeine from Coffee By-Products: A Review on Skincare Applications. Cosmetics.

[48] Jesus F., Gonçalves A.C., Alves G., Silva L.R. (2019). Exploring the Phenolic Profile, Antioxidant, Antidiabetic and Anti-Hemolytic Potential of *Prunus avium* Vegetal Parts. Food Res. Int..

[49] Lenchyk L. (2015). Determination of Phenolic Compounds in Prunus Domestica Leaves Extract. Scr. Sci. Pharm..

[50] Liaudanskas M., Viškelis P., Raudonis R., Kviklys D., Uselis N., Janulis V. (2014). Phenolic Composition and Antioxidant Activity of Malus Domestica Leaves. Sci. World J..

[51] Gervasi T., Calderaro A., Barreca D., Tellone E., Trombetta D., Ficarra S., Smeriglio A., Mandalari G., Gattuso G. (2022). Biotechnological Applications and Health-Promoting Properties of Flavonols: An Updated View. Int. J. Mol. Sci..

[52] Cao J., Jiang Q., Lin J., Li X., Sun C., Chen K. (2015). Physicochemical Characterisation of Four Cherry Species (*Prunus* spp.) Grown in China. Food Chem..

[53] Li X., Liu J., Chang Q., Zhou Z., Han R., Liang Z. (2021). Antioxidant and Antidiabetic Activity of Proanthocyanidins from Fagopyrum Dibotrys. Molecules.

[54] Bladé C., Arola L., Salvadó M.J. (2010). Hypolipidemic Effects of Proanthocyanidins and Their Underlying Biochemical and Molecular Mechanisms. Mol. Nutr. Food Res..

[55] Nunes M.A., Pimentel F., Costa A.S.G., Alves R.C., Oliveira M.B.P.P. (2016). Cardioprotective Properties of Grape Seed Proanthocyanidins: An Update. Trends Food Sci. Technol..

[56] Chen J., Chen Y., Zheng Y., Zhao J., Yu H., Zhu J. (2022). Relationship between Neuroprotective Effects and Structure of Procyanidins. Molecules.

[57] Nawrot-Hadzik I., Matkowski A., Kubasiewicz-Ross P., Hadzik J. (2021). Proanthocyanidins and Flavan-3-Ols in the Prevention and Treatment of Periodontitis—Immunomodulatory Effects, Animal and Clinical Studies. Nutrients.

[58] Alves-Santos A.M., Sugizaki C.S.A., Lima G.C., Naves M.M.V. (2020). Prebiotic Effect of Dietary Polyphenols: A Systematic Review. J. Funct. Foods.

[59] Qi Q., Chu M., Yu X., Xie Y., Li Y., Du Y., Liu X., Zhang Z., Shi J., Yan N. (2023). Anthocyanins and Proanthocyanidins: Chemical Structures, Food Sources, Bioactivities, and Product Development. Food Rev. Int..

[60] Khanam S., Mishra D.A., Shahid A., Pujari N.M. (2022). Therapeutic Indication of Phloridzin: A New Gleam for Metabolic Disorders. Phytomed. Plus.

[61] Khalid S., Bader H., Ain U. (2018). A Review on the Pharmacological Importance of Phloridzin and Its Conjugated Analogues. Pharmacologyonline.

[62] Guyot S., Serrand S., Le Quéré J.M., Sanoner P., Renard C.M.G.C. (2007). Enzymatic Synthesis and Physicochemical Characterisation of Phloridzin Oxidation Products (POP), a New Water-Soluble Yellow Dye Deriving from Apple. Innov. Food Sci. Emerg. Technol..

[63] Anunciato Casarini T.P., Frank L.A., Pohlmann A.R., Guterres S.S. (2020). Dermatological Applications of the Flavonoid Phloretin. Eur. J. Pharmacol..

[64] Liebelt D.J., Jordan J.T., Doherty C.J. (2019). Only a Matter of Time: The Impact of Daily and Seasonal Rhythms on Phytochemicals. Phytochem. Rev..

[65] Mohammadi Bazargani M., Falahati-Anbaran M., Rohloff J. (2021). Comparative Analyses of Phytochemical Variation within and between Congeneric Species of Willow Herb, *Epilobium hirsutum* and *E. parviflorum*: Contribution of Environmental Factors. Front. Plant Sci..

[66] Häkkinen S.H., Törrönen A.R. (2000). Content of Flavonols and Selected Phenolic Acids in Strawberries and *Vaccinium* Species: Influence of Cultivar, Cultivation Site and Technique. Food Res. Int..

[67] Generalić Mekinić I., Šimat V., Ljubenkov I., Burčul F., Grga M., Mihajlovski M., Lončar R., Katalinić V., Skroza D. (2018). Influence of the Vegetation Period on Sea Fennel, *Crithmum maritimum* L. (Apiaceae), Phenolic Composition, Antioxidant and Anticholinesterase Activities. Ind. Crops Prod..

[68] Deng L.Z., Xiong C.H., Pei Y.P., Zhu Z.Q., Zheng X., Zhang Y., Yang X.H., Liu Z.L., Xiao H.W. (2022). Effects of Various Storage Conditions on Total Phenolic, Carotenoids, Antioxidant Capacity, and Color of Dried Apricots. Food Control.

[69] Scibisz I., Mitek M. (2009). Effect of processing and storage conditions on phenolic compounds and antioxidant capacity of highbush blueberry jams. Pol. J. Food Nutr Sci..

[70] Nayak B., Liu R.H., Tang J. (2015). Effect of Processing on Phenolic Antioxidants of Fruits, Vegetables, and Grains—A Review. Crit. Rev. Food Sci. Nutr..

[71] Li W., Hydamaka A.W., Lowry L., Beta T. (2009). Comparison of Antioxidant Capacity and Phenolic Compounds of Berries, Chokecherry and Seabuckthorn. Cent. Eur. J. Biol..

[72] Prior R.L., Wu X., Schaich K. (2005). Standardized Methods for the Determination of Antioxidant Capacity and Phenolics in Foods and Dietary Supplements. J. Agric. Food Chem..

[73] Schlesier K., Harwat M., Böhm V., Bitsch R. (2002). Assessment of Antioxidant Activity by Using Different in Vitro Methods. Free Radic. Res..

[74] Olszewska M.A., Michel P. (2009). Antioxidant Activity of Inflorescences, Leaves and Fruits of Three Sorbus Species in Relation to Their Polyphenolic Composition. Nat. Prod. Res..

[75] Dziadek K., Kopeć A., Tabaszewska M. (2019). Potential of Sweet Cherry (*Prunus avium* L.) by-Products: Bioactive Compounds and Antioxidant Activity of Leaves and Petioles. Eur. Food Res. Technol..

